# Natural history of intraosseous low-grade chondroid lesions of the proximal humerus

**DOI:** 10.3389/fonc.2023.1200286

**Published:** 2023-08-11

**Authors:** Christopher M. LaPrade, Logan M. Andryk, Joshua L. Christensen, John C. Neilson, Adam N. Wooldridge, Donald A. Hackbarth, Manpreet Bedi, David M. King

**Affiliations:** ^1^ Department of Orthopaedic Surgery, Stanford University, Stanford, CA, United States; ^2^ Department of Orthopaedic Surgery, Medical College of Wisconsin, Milwaukee, WI, United States; ^3^ Department of Radiation Oncology, Medical College of Wisconsin, Milwaukee, WI, United States

**Keywords:** cancer/tumors, clinical outcomes, diagnostic imaging, shoulder, chondroid

## Abstract

**Introduction:**

Enchondromas and grade 1 chondrosarcomas are commonly encountered low-grade chondroid tumors in the proximal humerus. While there is a concern for malignant transformation, few studies have evaluated the natural history of these lesions. The purpose of this study is to evaluate the natural history of proximal humerus low-grade chondroid lesions managed both conservatively and surgically, and to define management criteria using clinical and radiographic findings for these low-grade chondroid lesions.

**Methods:**

The patient population included 90 patients intended for conservative treatment and 22 patients proceeding directly to surgery. Data collection was based on a combination of chart review and patient imaging and descriptive statistics were calculated for each group.

**Results:**

No malignant transformations were noted amongst any group. In the conservative treatment group, 7 of 64 (11%) progressed to surgery after an average of 20.3 months of conservative treatment due to persistent pain unexplained by other shoulder pathology. Importantly, 71% experienced continued pain at a mean of 53.1 months post-operatively. The group that went directly to surgery also demonstrated pain in 41% at an average follow-up of 57.3 months.

**Discussion:**

Low-grade cartilaginous lesions of the proximal humerus without concerning imaging findings can be managed with conservative treatment and the risk of malignant transformation is very low. Patients with a clear source of their shoulder pain unrelated to their tumor and without concerning characteristics on imaging can be managed with serial annual radiographic imaging. Patients undergoing surgery for these indolent tumors are likely to experience persistent pain even after surgery.

## Introduction

Enchondromas (ECs) and low-grade (grade 1) chondrosarcomas (CSs) are commonly encountered low-grade chondroid tumors. Enchondromas are benign cartilage tumors reported to account for 20% of all bone tumors in a series of 3,607 bone tumors (1), and are likely underreported due to many ECs being asymptomatic (1–5). Chondrosarcomas are malignant cartilage tumors and were reported to account for 37% of malignant bone tumors when including low- and high-grade CSs (1).

Distinguishing between an EC and low-grade CS is a difficult endeavor for orthopaedic surgeons, pathologists, and radiologists; however, it is an important one because these low-grade chondroid tumors are typically managed differently. EC lesions are often incidentally identified on imaging, with characteristics of minimal or absent endosteal scalloping or surrounding bone and soft tissue edema, as well as absent cortical breakthrough or soft tissue masses (1, 2, 6–9). Histologically, low-grade CSs show slightly more cellularity and myxoid changes than ECs, but accurate diagnosis remains challenging (8). Asymptomatic enchondromas throughout the body can be managed conservatively with serial imaging, while symptomatic tumors may require curettage. Additionally, one study suggested that tumors greater than seven centimeters in one dimension may require surgical curettage as the risk of malignant transformation is increased in these larger lesions (1). As opposed to ECs, low-grade CSs are often characterized by pain and more aggressive imaging findings including endosteal scalloping, surrounding marrow, or soft tissue edema. Low-grade CS’s typically require surgical treatment ranging from intralesional excision and curettage to wide excision depending on location (1, 2, 6–8, 10–13). While many low-grade chondroid lesions are managed without surgical intervention, there is a worry of malignant transformation into higher-grade tumors, with studies reporting a transformation rate of ECs ranging from 2% to 4% (1, 4, 10).

Low-grade chondroid tumors may develop anywhere in the skeleton, though they are commonly found in the proximal humerus, with Hong et al. (14) reporting that 2% of shoulder Magnetic Resonance Imaging (MRI) studies demonstrated an incidental proximal humerus EC, while Woltsche et al. (15) showed an EC prevalence of 0.39% in 21,550 patients receiving shoulder MRIs. In addition, studies have reported that 18-28% of ECs and 14% of low-grade CSs are located in the humerus (1, 3, 16). While multiple studies have advocated that low-grade chondroid lesions throughout the body may be managed conservatively with observation through serial imaging (1, 2, 6, 7, 12, 13, 17), there is a paucity of studies examining the natural history of these lesions, particularly in the proximal humerus.

The purpose of this study is to examine the natural history of proximal humerus low-grade chondroid lesions managed both conservatively and surgically at a single academic institution. The aim was to better define management criteria using clinical and radiographic findings for these low-grade chondroid lesions that are often discovered incidentally. We hypothesize that the vast majority of incidentally noted low-grade chondroid lesions in the proximal humerus can be observed and have a minimal risk of malignant transformation in the short-term follow-up period.

## Materials and methods

**Figure 1:**
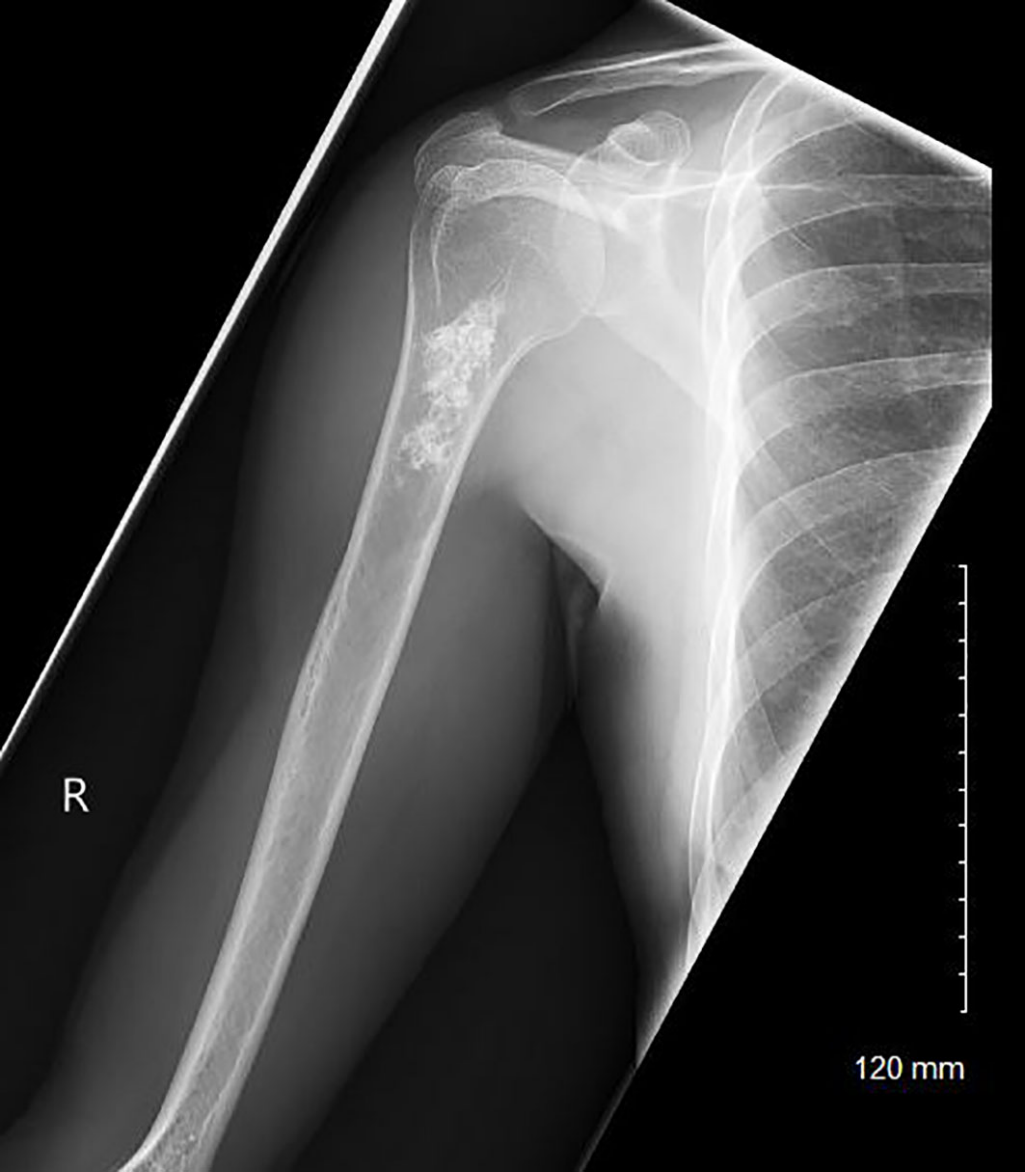
A radiograph of a patient with a low-grade chondroid lesion of the metadiaphyseal region of her right proximal humerus.

This was a level III retrospective cohort study of patients seen by four orthopaedic oncologists at a single academic institution from 2000 to 2022. Following Institutional Review Board approval, all patients with diagnoses of ECs or CSs (low- and high-grade) of the proximal humerus were identified (n=119) (**Figure 1**). All patients with high-grade, grade 2 or 3 CSs (n=1, grade 2) and multiple hereditary enchondromatosis (n=2, one grade 2 CS and one EC) were excluded to yield a study population of 116 ECs and grade 1 CSs (**Table 1**). All patients underwent a diagnostic evaluation, which included a clinical examination and diagnostic imaging (radiographs and axial imaging, consisting of either computed tomography (CT) or magnetic resonance imaging (MRI)).

**Table 1:** Demographics and lesion characteristics for each group of patients.

Demographics	Conservatively Managed	Conservative Group to Surgery	Surgically Managed
Number of Patients (Total = 86)	57	7	22
Age (years ± SD)	52.8 ± 10.4	36.4 ± 11.6	39.2 ± 15.4
Sex (M/F)	17 M, 40 F	1 M, 6 F	5 M, 17 F
Mean Length of Follow-up (months ± SD)	55.3± 45.3	54.3 ± 32.2 (post-surgery)	57.3 ± 47.8 (post-surgery)
Mean Length of Conservative Treatment (months ± SD)	N/A	20.3 ± 18.9	N/A

Imaging Findings	Conservatively Managed	Conservative Group to Surgery	Surgically Managed
Size at Initial Presentation (mm ± SD)	46.8 ± 30.0	34.8 ± 25.6	53.4 ± 34.8
Size on Follow Up (mm ± SD)	48.3 ± 31.1	N/A	N/A
Number of Lesions > than 7 cm	16%	14%	23%
Location of Lesion	28 M/D, 25 M, 3 D, 1 M/E	1 M/D, 4 M, 1 D, 1 M/E	10 M/D, 6 M, 3 D, 3 M/E
Mineralization	82%	43%	73%
Endosteal Scalloping	14%	14%	50%
Surrounding Bone Edema	9%	0%	23%
Surrounding Soft Tissue Edema	2%	0%	9%
Soft Tissue Mass	0%	0%	0%

Clinical Symptoms	Conservatively Managed	Conservative Group to Surgery	Surgically Managed
Shoulder Pain	79%	100%	91%
Pain Attributed to Tumor	0%	57%	68%
Night Pain	28%	43%	14%
Activity-Related pain	68%	100%	82%
Additional Shoulder Injury	79%	71%	36%
Concurrent Rotator Cuff Injury	63%	57%	27%
Biopsy Performed	4%	86%	91%
Incidentally Found	96%	71%	50%

M/D, metaphyseal/diaphyseal; M, metaphyseal; D, diaphyseal; M/E, metaphyseal/epiphyseal. N/A, Not applicable.

Data collection was based on a combination of chart review, imaging reports, and the original radiographs, CTs, and MRIs. The length of follow-up period was calculated from the first documentation of the lesion on imaging to the last follow-up radiograph for each patient. The size of the lesions at initial and final follow-up was determined by measuring the lesion using the Picture Archiving and Communication System (PACS) (McKesson Corporation, San Francisco, CA) at the maximal distance in the proximal/distal direction. The measurement was made in parallel to the cortex of the diaphysis, and measurements were confirmed with the imaging report in the patient record if it was available. Endosteal scalloping, surrounding bone and soft tissue edema, and the presence or absence of soft tissue masses were obtained from CT or MRI on the initial presentation of the patient. Other clinical information was collected from clinic notes. Tumors were considered incidentally found if other plausible explanations for the patient’s symptoms were identified on physical exam or imaging.

Data collection was performed using Microsoft Excel (Microsoft, Redmond, WA). Descriptive statistics were calculated for each of the final groups, with means and standard deviations calculated for size, age, follow-up time, and time to surgery. All other characteristics were reported as percentages of the overall group data. Fisher’s Exact Testing was used to generate statistical significance for various qualitative characteristics of biopsy-proven ECs versus CS, while t-testing was utilized for quantitative characteristics such as lesion size. The statistical significance threshold was set at p<0.05.

## Results

**Figure 2:**
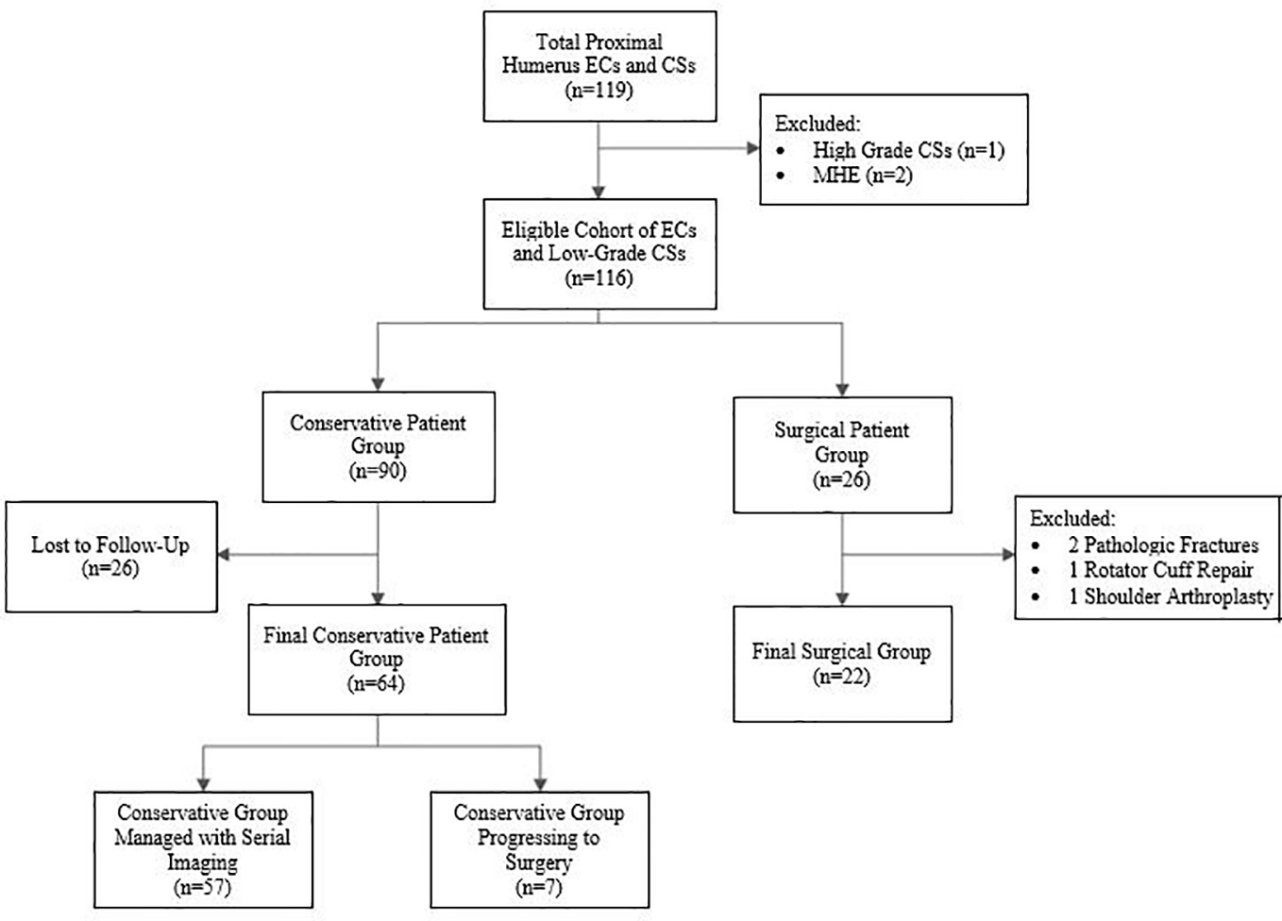
Flow chart of the inclusion and exclusion criteria for the final patient groups. ECs, enchondromas; CSs, chondrosarcomas; MHE, Multiple Hereditary Enchondromatosis.

The study population was divided into two groups: patients intended for conservative treatment (n=90) and patients proceeding directly to surgery (n=26) (**Figure 2**). The conservative patient group consisted of patients who were originally intended for observation of the tumors with serial radiographic imaging at 3 months, 6 months and then yearly, per the guidelines established by Marco et al. (7). Patients without at least 3 months of follow-up with radiographs were excluded (n=26), leaving 64 in the final group intended for conservative treatment. The patients that proceeded directly to surgery included patients who displayed concerning radiographic findings or clinical findings in which conservative treatment was not recommended, such as in patients with evidence of endosteal scalloping or surrounding bone or soft tissue edema. To evaluate a more homogenous patient population, four of the original 26 patients in the surgical treatment group were excluded from the current study because the surgeries were performed for reasons unrelated to the concern for malignancy or symptoms from the lesion. These surgeries included two open reduction and internal fixation procedures for pathologic fractures, one rotator cuff repair with intended anchor placement over the tumor location, and one shoulder arthroplasty (**Figure 2**).

**Table 2** contrasts clinical and radiographic characteristics of ECs and low-grade CSs that were confirmed on pathology or biopsy. Overall, 79% of low-grade chondroid tumors in the proximal humerus were incidentally found secondary to work-up for other shoulder pathology (**Table 3**).

**Table 2:** Clinical and radiographic characteristics of ECs and low-grade CSs that were confirmed on pathology or biopsy.

Demographics	EC/Low Grade Cartilaginous Lesions	Grade 1 CSs	p-Value
Patients in Each Group (Total = 28)	23	5	
Age (years ± SD)	39.7 ± 12.3	48.4 ± 9.0	0.183
Sex (M/F)	19 F, 4 M	5 F	0.568
Patients with Surgical Intervention	91%	100%	1
Time of Progression to Surgery (months ± SD)	8.7 ± 13.8	1.7 ± 1.2	0.289

Imaging Findings	EC/Low Grade Cartilaginous Lesions	Grade 1 CSs	p-Value
Size at Initial Presentation (mm ± SD)	45.3 ± 34.6	59.5 ± 20.3	0.441
Number of Lesions > 7 cm	22%	20%	1
Location of Lesion	9 M, 8 M/D, 4 D, 2 E/M	3 M/D, 2 M	
Mineralization	61%	100%	0.144
Endosteal Scalloping	35%	60%	0.353
Surrounding Bone Edema	17%	40%	0.286
Surrounding Soft Tissue Edema	9%	20%	0.459
Soft Tissue Mass	0%	0%	1

Clinical Symptoms	EC/Low Grade Cartilaginous Lesions	Grade 1 CSs	p-Value
Shoulder Pain	91%	100%	1
Pain attributed to tumor	52%	100%	0.125
Night Pain	35%	0%	0.281
Activity-Related pain	87%	80%	1
Additional Shoulder Injury	43%	40%	1
Concurrent Rotator Cuff Injury	30%	40%	1
Incidentally noticed?	65%	40%	0.353

M/D, metaphyseal/diaphyseal; M, metaphyseal; D, diaphyseal; M/E, metaphyseal/epiphyseal.

**Table 3:** Incidentally noted low-grade proximal humerus cartilaginous lesions.

Incidentally Noted ECs and Grade 1 CSs	Number of Patients	%
Total Patients	86	
# Incidentally Noted	68	79%
XR for Suspected Rotator Cuff Injury	15	17%
Bone Scan	10	12%
XR after Trauma	10	12%
Chest XR	8	9%
MRI for Suspected Rotator Cuff Injury	7	8%
MRI after Trauma	3	3%
MRI for Lipoma/Lump in Shoulder	3	3%
MRI for Cervical Radiculopathy/Arthritis	2	2%
XR Decreased ROM/Frozen Shoulder	2	2%

Methodology was only included if it found more than one lesion. XR, x-ray; MRI, magnetic resonance imaging; ROM, range of motion.

### Conservative treatment group

64 patients were initially conservatively managed (**Figure 2**, **Table 1**). 57 of these patients (89%) continued with conservative treatment and did not progress to surgery, and the average follow-up was 55.3 months. Average size of the 57 lesions at initial identification was 4.68 ± 3.0cm, while average size at most recent radiologic follow-up was 4.83 ± 3.11cm. 9/57 lesions (18%) were larger than 7cm in length on initial finding. Almost all lesions remained stable in length at final follow-up, with only 4/57 (7%) total lesions increasing in length by ≥1cm at an average of 105.25 months of follow-up, and only 1/9 (11%) lesions >7cm increasing in length. None of these conservatively managed lesions were identified to have radiologic characteristics suggestive of transformation to a more malignant state over the course of their care.

Seven patients originally intended for conservative management (11%) progressed to surgery after a mean conservative treatment time of 20.3 months. All 7 underwent curettage with bone grafting, with 3/7 involving prophylactic plating. At a mean of 53.1 months of follow-up, 5 of the 7 surgical patients (71%) experienced persistent pain, but there were no other notable post-operative complications noted. Indications (some patients had multiple) for eventual surgical intervention were persistent pain (100%) and growth of tumor (29%). All lesions were diagnosed as enchondromas or well differentiated chondroid lesions following pathology review, except for one patient whose pathology data was inaccessible. Further evaluation of these 7 tumors did not reveal any malignant transformation to intermediate or high-grade CSs.

### Surgical treatment group

The surgical treatment group consisted of 22 patients indicated for surgery without preceding conservative treatment. Indications for surgery (some patients had multiple) were pain (59%), concern for CS or CS identified on biopsy (40%), endosteal scalloping (9%), lesion breaching the majority of the cortex (5%), and concern for chondroblastoma (5%).

**Figure 3:**
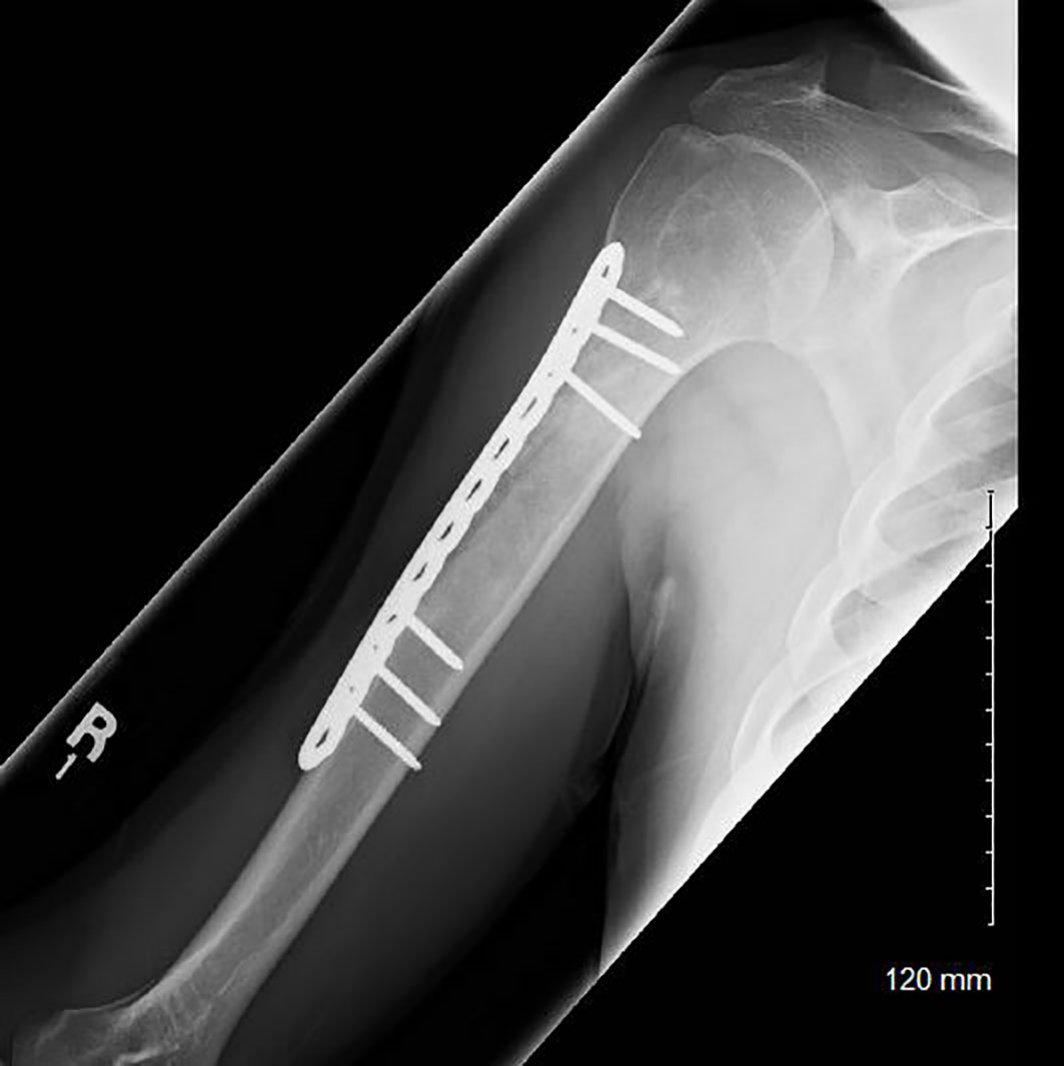
Post-operative radiograph of a patient who underwent curettage, grafting, and plating of a low-grade chondroid lesion of his proximal humerus.

All 22 patients were surgically treated with curettage and bone grafting (**Figure 3**), 9 of which also involved prophylactic plating. At an average follow-up of 57.3 months, 41% of patients noted continued pain at their most recent follow-up. Four post-operative complications were noted (18%): 2 patients required hardware removal, 1 patient experienced post-operative adhesive capsulitis, and 1 patient experienced a pathologic fracture through the curettage site secondary to multiple post-operative falls. Pathology noted 15 ECs or well-differentiated cartilaginous lesions (68%) and 5 low-grade CSs (23%). Pathology results were not available for two patients. At most recent follow-up, no tumor recurrences or malignant transformations were identified in the surgical treatment group.

### Biopsy-proven lesions

28 of the 90 patients met inclusion criteria and had lesions with a biopsy-proven diagnosis. 8 biopsies were performed prior to a potential surgery (4 open core biopsies and 4 image-guided percutaneous core biopsies), and 20 were sent for pathologic diagnosis at the time of operative intervention. 23 of the biopsied patients (82%) were diagnosed with ECs or well-differentiated cartilaginous lesions, while 5 patients (18%) were diagnosed with low-grade CSs. While more patients with biopsy-proven CSs showed radiologic characteristics like endosteal scalloping, mineralization, and surrounding bone or soft tissue edema, there were no statistically significant differences between the two types of lesions.

## Discussion

This study was a retrospective review of 86 patients with low grade chondroid lesions of the proximal humerus, and none of the 86 lesions ultimately transformed to a more malignant state. Given the exceedingly low risk of malignant transformation combined with the long-term stability of these lesions and low potential for significant growth (exemplified by only 7% of conservatively managed lesions increasing in length by >1cm at final multi-year follow-up), we believe that the majority of low-grade chondroid lesions in the proximal humerus should be initially managed with conservative treatment and serial radiographic imaging. Although patients may experience small growth of their lesion over time, many remain asymptomatic and do not show other concerning findings for malignant transformation on imaging. Because of this relative stability, we believe that small growth of lesions without development of significant symptoms or other concerning imaging findings is not a direct indication for surgical intervention. Similarly, while a length >7 cm has previously been cited as a surgical indication due to concerning potential for malignant transformation (1), our study showed continued stability of almost all lesions over this size and we do not believe that this alone should warrant surgery without the presence of significant symptoms or additional concerning imaging findings.

There was a small percentage (11%) of patients initially indicated for conservative management who progressed to surgery after an average of 20.3 months; however, none of these patients had malignant transformation of their lesions and the majority progressed to surgery because of persistent pain. This is consistent with one other study (18) which reported a 16% surgical rate following initial conservative treatment of cartilaginous tumors of the long bones. While pain was often our primary surgical indication, 71% of these patients still experienced persistent post-operative pain at an average of 53.1 months. While multiple studies have proposed that low-grade chondroid lesions throughout the body may be managed conservatively with serial imaging (1, 2, 6, 7, 12, 13, 19), we believe our current study is one of the first to follow these patients with proximal humerus tumors in the short-term to determine the possibility of malignant transformation or failure of conservative treatment.

Few studies have evaluated the clinical outcomes after surgical intervention of primary bone tumors in the proximal humerus, and many of these studies focus on higher-grade tumors, including osteosarcomas, Ewing’s sarcomas, giant cell tumors, and chondrosarcomas, which are often treated with allograft implants (20–23). One study highlighting atypical cartilaginous tumors of the proximal humerus (24) showed that resection and cementing of these lesions led to excellent patient satisfaction and low post-operative pain, though another study by the same author (25) showed that surgery did not provide superior outcomes to clinical observation for cartilaginous tumors of long bones. The latter study appears to be more consistent with our study findings, as many patients achieved excellent outcomes with clinical observation and serial radiographic follow-up. In our study, 26% of our final patient population went directly to surgery for their low-grade proximal humerus cartilaginous lesions. In comparison to the conservative group, these patients had higher rates of concerning findings on imaging, including endosteal scalloping (50% versus 14%), surrounding bone edema (23% versus 9%), and size greater than 7cm (23% versus 16%). Despite these concerning imaging features, only 23% of the surgical group demonstrated low-grade CSs on pathology.

At a mean follow-up of 57.3 months post-operatively, the immediate surgical group also demonstrated persistent pain in 41% of patients and complications in 18%. The group of patients who were originally intended for conservative management but progressed to surgery also experienced persistent pain in 71%; however no notable complications were experienced in these patients. We hypothesize that the reports of high post-operative pain are at least partially related to concomitant shoulder pathology in the patients, which were present in 45% of the entire surgical population. This hypothesis is consistent with the findings of other studies (6, 25) which show that ECs usually present in conjunction with additional shoulder pathology.

Currently, literature has reported that certain clinical and radiographic criteria may help in distinguishing between ECs and CSs; nevertheless, there is controversy and most studies have not distinguished between low- and high-grade CSs. In this study, **Table 2** illustrates the different characteristics of ECs and low-grade CSs which were confirmed on pathology or biopsy. Qualitatively, albeit with a small sample size, our data indicated ECs are more common than low-grade CSs and may be less likely to present with concerning findings on imaging, such as a larger size, endosteal scalloping, mineralization, or surrounding bone or soft tissue edema. These findings do correlate with the 2020 WHO classification of bone tumors (8) and previous studies reporting that CSs may be significantly increased in size in comparison to ECs (3, 16). Murphey et al. (16) also reported that CSs (low- and high-grade were not differentiated) displayed deeper and more extensive scalloping, pathologic fracture, more cortical destruction, and presence of soft tissue masses. However, it should be noted that a more recent study reported a low reliability in differentiating benign from malignant cartilaginous tumors, even for experienced radiologists and pathologists (12).

The presence of pain has often been cited as an indicator of CS in comparison to ECs, but this is controversial and some studies have shown significant differences in pain between the two lesions (16, 26, 27) while others have not (2). In one of the few studies comparing low-grade CSs to ECs, Welkering et al. (27) reported that low-grade CSs demonstrated significantly increased pain compared to ECs. Qualitatively in our study, pain was similar for these low-grade lesions that underwent surgery or biopsy (91% and 100% for ECs and low-grade CSs, respectively). However, our conservative treatment group, whose lesions were infrequently biopsied and theoretically would be more likely to be of a lower grade, did show decreased overall pain (79%) as compared to the surgical group.

This study has some notable limitations. Importantly, 29% of patients did not receive follow-up imaging at 3 months following the initial visit. Many of these patients who did not follow up may have been initially referred for clearance to undergo other shoulder procedures. It is likely that a number of these patients had routine follow up with their referring provider following surgery and were not sent back to us given the likely stability of these lesions as demonstrated in this study. While this is a reasonably large group of patients, we believe that many of these patients ultimately had incidentally found low-grade chondroid lesions which were asymptomatic and did not require long-term follow-up. All patients progressing with conservative treatments were informed that their lesions were likely to be benign, but the potential for malignant transformation was discussed with all patients. As the primary musculoskeletal oncology providers in the region, we would have expected to see any patients lost to follow-up whose lesions would have ultimately become symptomatic and transformed to higher-grade lesions. Additionally, our study was a short-term follow-up of patients with low-grade cartilaginous lesions, and we cannot use the lack of malignant transformation to assume that these lesions will be stable in the long-term follow-up period. Our mean follow-up for the conservative group was 55.3 months, and our final study group consisted of all patients who returned for at least one radiographic follow-up at only 3 months in order to get the most accurate sense of how many patients would fail conservative treatment. We therefore recommend further studies to explore the stability of these lesions in the long-term.

### Recommendations

**Figure 4:**
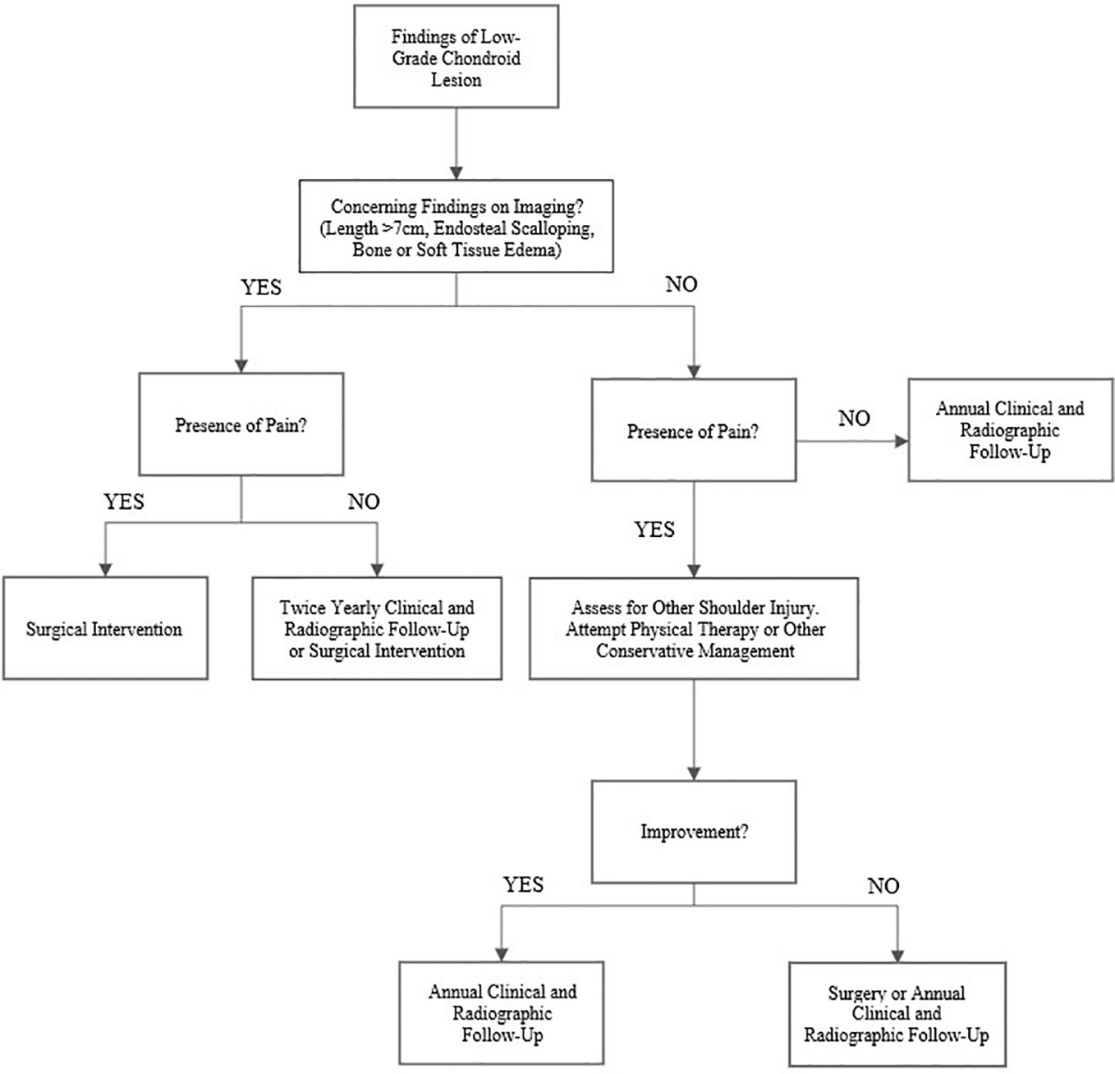
Proposed treatment algorithm for low-grade chondroid tumors in the proximal humerus. In general, we would recommend that after 2 to 3 years of yearly radiographic follow-up without significant change and a lack of concerning findings on imaging, patients should be instructed to present to clinic as needed if their pain increases.

Based on our results, we believe there is a clear group of patients who do not require surgical intervention for low-grade chondroid lesions. We recommend a treatment algorithm as shown in **Figure 4**. Those patients with a clear source of their shoulder pain unrelated to their tumor and without concerning radiologic characteristics, such as endosteal scalloping or bone or soft tissue edema, likely will not benefit from surgical intervention in the short-term. In these lesions without concerning imaging findings, consideration should also be taken regarding whether a biopsy is truly necessary. Given the stability and low risk nature of these low-grade chondroid lesions, biopsy is often not necessary and may increase the risk for complications on its own (28–30). As the lesion is very likely to remain stable overall, we would instruct these patients to follow-up clinically for yearly physical examination with radiographs, with the caveat that they should present for further evaluation if their pain significantly increases. In general, we would recommend that after 2 to 3 years of annual radiographic follow-up without significant change and a lack of concerning features on imaging, patients should present to clinic as needed if they experience new or increasing symptoms. We would also recommend initiating physical therapy or offering steroid injection under fluoroscopic guidance for patients with continued pain, especially in those with an additional unrelated shoulder injury identified on imaging or clinical exam.

For those patients with pain and potentially concerning imaging findings such as significant endosteal scalloping or bone or soft tissue edema, we would recommend proceeding to surgery directly as their lesion is more likely to be contributing directly to their pain. This is particularly true for patients without evidence of other concomitant shoulder pathology such as rotator cuff injury. Although surgical curettage and grafting may help with overall symptoms, it is important to communicate that there is a high likelihood that patients will still experience some level of persistent pain post-operatively. Asymptomatic patients with incidentally noted lesions that have concerning features on imaging may attempt conservative treatment, but these patients should have more frequent clinical and radiographic follow-up to ensure that their lesions do not progress to a more malignant state.

## Conclusion

Most low-grade cartilaginous lesions of the proximal humerus can be managed with conservative treatment. Patients who present with more concerning findings such as larger lesion size, endosteal scalloping, persistent pain, the presence of bone or soft tissue edema, or a lack of another explanation for their pain may be considered for surgical curettage. We propose that patients with a clear source of their shoulder pain unrelated to their tumor and without concerning characteristics on imaging likely do not require surgical intervention in the short-term and can be managed with serial annual radiographic imaging. In the absence of significant pain or functional changes, given the low risk of malignant conversion of these lesions, patients do not require persistent follow-up imaging following demonstrated imaging stability documented over 2-3 years. Lastly, we would counsel patients with significant pain that surgery may not completely alleviate their pain post-operatively.

## Data availability statement

The raw data supporting the conclusions of this article will be made available by the authors, without undue reservation.

## Ethics statement

The studies involving human participants were reviewed and approved by Medical College of Wisconsin Institutional Review Board #5. Written informed consent from the participants’ legal guardian/next of kin was not required to participate in this study in accordance with the national legislation and the institutional requirements.

## Author contributions

JN, AW, DH, MB, and DK conceived and planned the research, and performed the patient care as described in the manuscript. CL, LA, and JC planned the project, carried out the retrospective review, and analyzed the data. CL and LA took the lead in writing the manuscript, while all authors provided critical feedback and helped shape the final research project and manuscript. All authors interpreted and analyzed the results. All authors contributed to the article and approved the submitted version.
